# [^18^F]TFB PET/CT misses intense [^124^I]iodine-avid metastases after redifferentiation therapy in metastatic thyroid cancer

**DOI:** 10.1186/s13550-024-01138-x

**Published:** 2024-10-08

**Authors:** Philipp Backhaus, Keith S. Pentlow, Alan L Ho, Audrey Mauguen, James A Fagin, Naga Vara Kishore Pillarsetty, Serge K. Lyashchenko, Eva Burnazi, Ronald A. Ghossein, Shalini Chhabra, Murad Abusamra, Steven M. Larson, Heiko Schöder, Joseph O’Donoghue, Wolfgang Weber, Ravinder K. Grewal

**Affiliations:** 1 Molecular Imaging and Therapy Service, Department of Radiology, Memorial Sloan Kettering Cancer Center, 1275 York Ave, P.O. Box 77, New York, NY 10065 USA; 2 Department of Nuclear Medicine, University Hospital Münster, Münster, Germany; 3 Department of Medical Physics, Memorial Sloan Kettering Cancer Center, New York, NY USA; 4 Department of Medicine, Memorial Sloan Kettering Cancer Center, New York, NY USA; 5 Department of Radiology, Weill Cornell Medical College, New York, NY USA; 6 Department of Epidemiology & Biostatistics, Memorial Sloan Kettering Cancer Center, New York, NY USA; 7 Department of Pathology and Laboratory Medicine, Memorial Sloan Kettering Cancer Center, New York, NY USA; 8 Department of Nuclear Medicine, Technical University Munich, Munich, Germany

**Keywords:** Thyroid cancer, PET/CT, [^18^F]TFB, [^124^I]iodine, Redifferentiation

## Abstract

**Background:**

Fluorine 18-labelled tetrafluoroborate ([^18^F]TFB) is a substrate for the sodium/iodide symporter. In thyroid cancer, [^18^F]TFB-PET/CT may be an alternative to iodine imaging to evaluate the extent of disease, eligibility for radioiodine treatment, and success of redifferentiation therapies. We report the results of a pilot study to determine tumor uptake of [^18^F]TFB and compare its properties to [^124^I]IodinePET/CT in patients with metastatic thyroid cancer.

**Methods:**

Five patients were included in a prospective study. All patients received PET/CT 1 h after injection of 356 ± 12 MBq [^18^F]TFB and were given 230 ± 9 MBq [^124^I]Iodine orally on the same day, followed by PET/CT after 48 h. Before redifferentiation therapy, patients underwent an additional baseline [^124^I]Iodine PET/CT. Cases were analyzed by two board-certified specialists. Detection rates and Spearman correlation for [^18^F]TFB and [^124^I]Iodine were calculated.

**Results:**

Three patients had poorly differentiated thyroid cancer and received trametinib in a redifferentiation trial. Two patients had papillary thyroid cancer and did not receive redifferentiation therapy. Of the 33 lesions seen before/without redifferentiation therapy, 19 (58%) were visible on [^18^F]TFB and 30 (91%) on [^124^I]Iodine imaging. In the patients who underwent redifferentiation therapy, 48 lesions were newly seen on [^124^I]Iodine PET/CT with a median SUV_max_ of 3.3 (range, 0.4–285.0). All of these lesions were [^18^F]TFB-negative.

**Conclusion:**

[^18^F]TFB failed to predict radioactive iodine uptake in patients with poorly differentiated thyroid cancer who underwent redifferentiation therapy with trametinib. It is unclear whether such discrepancies may also occur in other redifferentiation therapies or may even be encountered in redifferentiation-naïve thyroid cancer.

**Trial registration number:**

NCT03196518, registered on June 22, 2017.

## Introduction

Iodine is central to the function of follicular thyroid cells that produce and store thyroid hormone. This function is usually sustained in differentiated thyroid cancer (DTC), enabling detection of cancer by molecular imaging with radioactive iodine and radioiodine therapy (RIT). However, DTC can develop de-differentiated metastases or be primarily diagnosed as poorly differentiated thyroid cancer, which is associated with worse outcomes, frequent refractoriness for RIT, and failure to localize disease with iodine imaging [1]. In this scenario, kinase inhibitors have been established as a novel treatment option [2]. Additionally, specific kinase inhibitors possess the ability to restore iodine uptake in dedifferentiated or poorly differentiated cancer [3], referred to as “redifferentiation.”

Following the concept of theranostics, iodine imaging in metastatic thyroid cancer has served a dual purpose, i.e., to establish the presence of and localize local or metastatic cancer as well as to evaluate patient eligibility for RIT. [^124^I]Iodine PET/CT scanning brings together the superior spatial resolution and quantification of PET/CT with a tracer biological equivalent to [^131^I]Iodine [4], allowing precise lesional and whole-body dosimetry if dynamic scanning is applied [5]. However, this approach is currently limited to specialized centers: on one hand, [^124^I]Iodine cannot be produced by all cyclotrons and multiple high energy gamma peaks complicate quantitative image reconstruction [6]. On the other hand, multi-timepoint scanning protocols and sophisticated analysis techniques are required for [^124^I]Iodine based dosimetry. Thus, single-timepoint single-photon imaging either with [^123^I]Iodine or [^131^I]Iodine is most commonly used to detect metastases and to prospectively or retrospectively evaluate the potential success of RIT.

The [^18^F]-labeled radiotracer tetrafluoroborate ([^18^F]TFB) is a specific substrate of the sodium iodine symporter (NIS) [7, 8] that was recently developed and evaluated in patients [9]. In thyroid cancer, [^18^F]TFB could offer the technical advantages of PET scanning while requiring less sophisticated production and imaging, with the added benefit of lower radiation burden than [^124^I]Iodine. However, [^18^F]TFB may not be a perfect equivalent of iodine, as it is (as [^99m^Tc] pertechnetate) a sole substrate of NIS, but not of downstream enzymes/proteins that are involved in processing and retaining iodine, as thyroperoxidase (TPO) or thyroglobulin (TG). Therefore, the adequacy of [^18^F]TFB as a functional mimic of iodine remains to be evaluated.

In this study, we report the results of the prospective pilot study “Study of PET Imaging with ^18^FTFB in Patients with Thyroid Cancer” (NCT03196518). Based on the results of five patients with either poorly differentiated thyroid cancer having undergone redifferentiation therapy or with papillary thyroid cancer, we assessed the detection capabilities of [^18^F]TFB compared to [^124^I]Iodine PET/CT.

## Materials and methods

### Patients

Five patients were enrolled in a prospective open-label, single-group clinical trial entitled “Study of PET Imaging with ^18^FTFB in Patients with Thyroid Cancer” to evaluate the safety and detection capability of [^18^F]TFB. The trial was registered on ClinicalTrials.gov (NCT03196518) and approved by the institutional review board of Memorial Sloan Kettering Cancer Center, New York, USA (IRB #17–315). Written informed consent was obtained from all patients. Inclusion criteria included confirmed thyroid carcinoma of follicular origin with radiographically evident disease, age ≥ 18, ECOG performance status ≤ 2, normal organ and bone marrow function, negative pregnancy test in women, consent to contraception, and ability to understand consent. Exclusion criteria included uncontrolled intercurrent illness, pregnancy, lactation, breastfeeding, inability to follow low-iodine diet or requiring high-iodine medication, and/or iodinated intravenous contrast within three months. All 5 enrolled patients were eligible and scanned at Memorial Sloan Kettering Cancer Center between 10/2019 and 02/2022. Patients 1–3 were also enrolled in the trametinib redifferentiation and [^124^I]Iodine/[^131^I]Iodine dosimetry trial (NCT02152995; IRB #13–157) and patients 4–5 were also enrolled on the [^124^I]Iodine dosimetry trial (NCT03647358; IRB #18–253) covering iodine scanning and dosimetry in these patients. No patient data from this study sample were previously published.

### Scanning and trial protocols

**Fig. 1:**
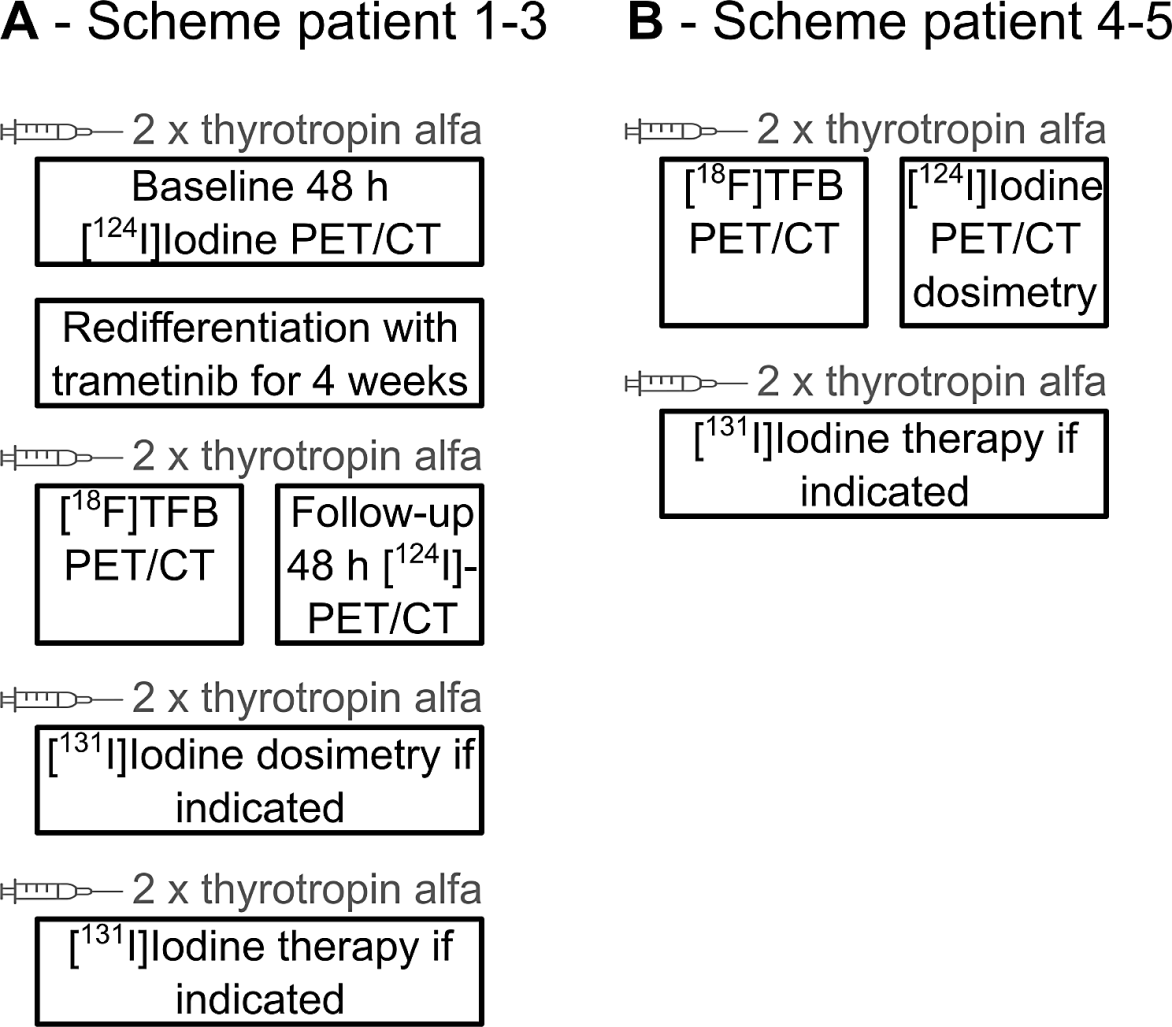
Scheme of diagnostic and therapeutic procedures comprising trials NCT02152995 and NCT03196518 and clinical care in patients 1–3 (**A**) and NCT03647358 and NCT03196518 and clinical care in patients 4–5 (**B**)

All patients followed a low-iodine diet for at least 7 days before any tracer application and were injected intramuscularly with 0.9 mg thyrotropin alfa (Thyrogen, Sanofi Genzyme, Paris, France) in the two consecutive days before any iodine or [^18^F]TFB application. All PET/CTs were conducted with arms down from the vertex to the upper thighs on Discovery 710 PET/CT systems (GE Healthcare, Chicago, IL). Non-contrast-enhanced low-dose companion CT was used for attenuation correction. Patients were planned to be injected with 185–370 MBq [^18^F]TFB (< 50 µg TFB) and scheduled to be scanned with PET/CT at 90 ± 30 min p.i. Patients were planned to receive 148–222 MBq of oral [^124^I]Iodine directly after TFB scanning followed by PET/CT 48 h after. Patients also enrolled on the [^124^I]Iodine dosimetry trial (NCT03647358, IRB #18–25) underwent additional [^124^I]Iodine scanning time points for dosimetry. Patients enrolled in the trametinib redifferentiation and [^124^I]Iodine/[131I]Iodine dosimetry trial (NCT02152995, IRB #13–157) underwent baseline and post-redifferentiation therapy [^124^I]Iodine PET. For safety assessment, patients were clinically monitored with vital signs before and two hours after tracer administration and had an additional visit two days after TFB administration. See Fig. 1 for an overview of trial procedures and clinical care.

### Image analysis and dosimetry

[^124^I]Iodine and [^18^F]TFB images were analyzed and subsequently verified by board-certified nuclear medicine specialists. Lesion SUV_max_ was obtained with spherical volumes of interest using GE AW PET VCAR (GE Healthcare, Chicago, IL) in [^18^F]TFB scans and [^124^I]Iodine scans two days after injection. For both PET tracers, lesions were considered positive if they demonstrated clear focal uptake above the background and noise level of the lung for lung metastases and above the background and noise level of blood for local relapse, nodal, and non-pulmonary distant metastases. Correlative diagnostic CT and [^18^F]Fluorodeoxyglucose (FDG)-PET/CT (if available) was integrated into the reading process to validate the plausibility of findings, but were not part of the formal analysis. No minimal spatial extent and no definite structural correlate was required in companion non-diagnostic low-dose-CT for foci to be considered, as long as the compartments showed other unequivocal sites of metastatic disease in correlative imaging. This could also include punctuate lung nodules.

### Radiochemistry

Synthesis of [^18^F]-NaBF4 ([^18^F]TFB): [^18^F]-NaBF4 was synthesized manually based on a previously published protocol by Doherty and colleagues [10]. Briefly, [^18^F]-Fluorine was loaded onto a Sep-Pak^®^ Light Waters Accell Plus™ QMA Cartridge preconditioned first with 5 mL of 0.9% sodium chloride (NaCl), then with 10 mL of water. [^18^F]-Fluorine was eluted from Sep-Pak^®^ Light Waters Accell Plus™ QMA Cartridge with 350 µL of 0.9% sodium chloride into the glass reaction vial containing 10 mg of 15-Crown-5 to 500 µL in acetonitrile. Water was removed by azeotropic distillation of the solvent mixture under the flow of Argon at 95 °C. After drying, 1 mL of acetonitrile was added and the solvent was evaporated at 95 °C under argon flow and the process was repeated two additional times. Boron trifluoride diethyl etherate (Et2OBF3, 2 µL [2.3 mg, 16.2 µmoles]) in acetonitrile (500 µL) was added to the vial containing [^18^F]-Fluorine and allowed to react for 10 min at 80 ºC followed by cooling for 3 min. The crude reaction mixture was passed through a Sep-Pak^®^ Light Alumina N cartridge 50–300 μm (preconditioned with 5 mL of ethanol followed by 10 mL of sterile water) into a vial containing 1 mL of sterile water for injection. This mixture was loaded on to a Sep-Pak^®^ Light Waters Accell Plus™ QMA Cartridge and rinsed with 10 mL sterile water. The final purified product was eluted from the Sep-Pak^®^ Light Waters Accell Plus™ QMA Cartridge using 2.5 mL of 0.9% sodium chloride injection, USP into the final product assembly via the sterilizing filter and was used for human studies. Quality control was performed on aliquots to confirm purity and identity.

### Statistics

Statistical tests were performed with Rv4.1.1. Continuous variables are described as median and range. Wilcoxon signed rank test was used to compare uptake in lesions between different scan techniques. Spearman’s correlation coefficient was calculated for lesions’ SUV_max_ of [^18^F]TFB and [^124^I]Iodine, with a 95% confidence interval (95% CI) using the method in Bonett and Wright [11]. To calculate correlation and enable visualization in log-log-plots, negative [^18^F]TFB or [^124^I]Iodine lesions’ SUV_max_ were set to the lowest detected SUV_max_ in visible lesions in the entire study cohort minus 0.1. The actual value used does not impact the correlation as the rank is used in the calculation, as long as it is the lowest value.

## Results

### Patient and scanning characteristics

Between 10/2019 and 02/2022, five patients were enrolled. No patient was excluded from the analysis. Patient characteristics at the timepoint of [^18^F]TFB scanning are listed in Table 1. Median age was 49 years (range, 27–66). All patients had a history of metastatic thyroid cancer and had previously undergone thyroidectomy and adjuvant [^131^I]Iodine therapy. Patients 1–3 had poorly differentiated thyroid cancer and underwent redifferentiation therapy (Fig. 1). Patients 4–5 had papillary thyroid cancer and did not undergo redifferentiation therapy. Patients were injected with 344–370 (median 359) MBq [^18^F]TFB intravenously and scanned 60–78 (median 60) min for 3 min per bed position afterwards. For near-simultaneous [^124^I]Iodine PET/CT, 218–243 (median 229) MBq [^124^I]Iodine were given orally 89–106 (median 95) min after the TFB application, and a PET/CT was performed 43.7–45.3 (median 44.7) hours later for 6.4–7.7 min per bed position. Patients 1–3 had previously undergone [^124^I]Iodine PET/CT prior to starting redifferentiation therapy 28 days before TFB scanning. These (baseline) scans were performed after oral administration of 231–238 MBq [^124^I]Iodine and scans were performed 46.6–48.4 h afterwards. These patients were treated with trametinib 2 mg daily for 4 weeks in between pre- and post-redifferentiation scanning.

**Table 1:** Patient characteristics at the timepoint of TFB scanning

	All patients (*n* = 5)
Age (years)	49 (27–66)
Weight (kg)	88 (65–101)
Male: female gender	2:3
Thyroid cancer subtype	
Poorly differentiated cancer	3
Papillary cancer	2
Baseline TG (ng/mL)	12.1 (0.3-8181.3)
Stimulated TG (ng/mL)	91.3 (10.1-11800.9)
Stimulated TSH (µIU/mL)	106.9 (43.7- 169.4)
Anti-TG	Positive in one patient with 4784.9 IU/mL
Creatinine (mg/dL)	0.8 (0.7–1.3)

Numbers are given as median and range

### Comparison of [^18^F]TFB and near-simultaneous [^124^I]iodine PET/CT in patients with or without redifferentiation therapy

**Fig. 2:**
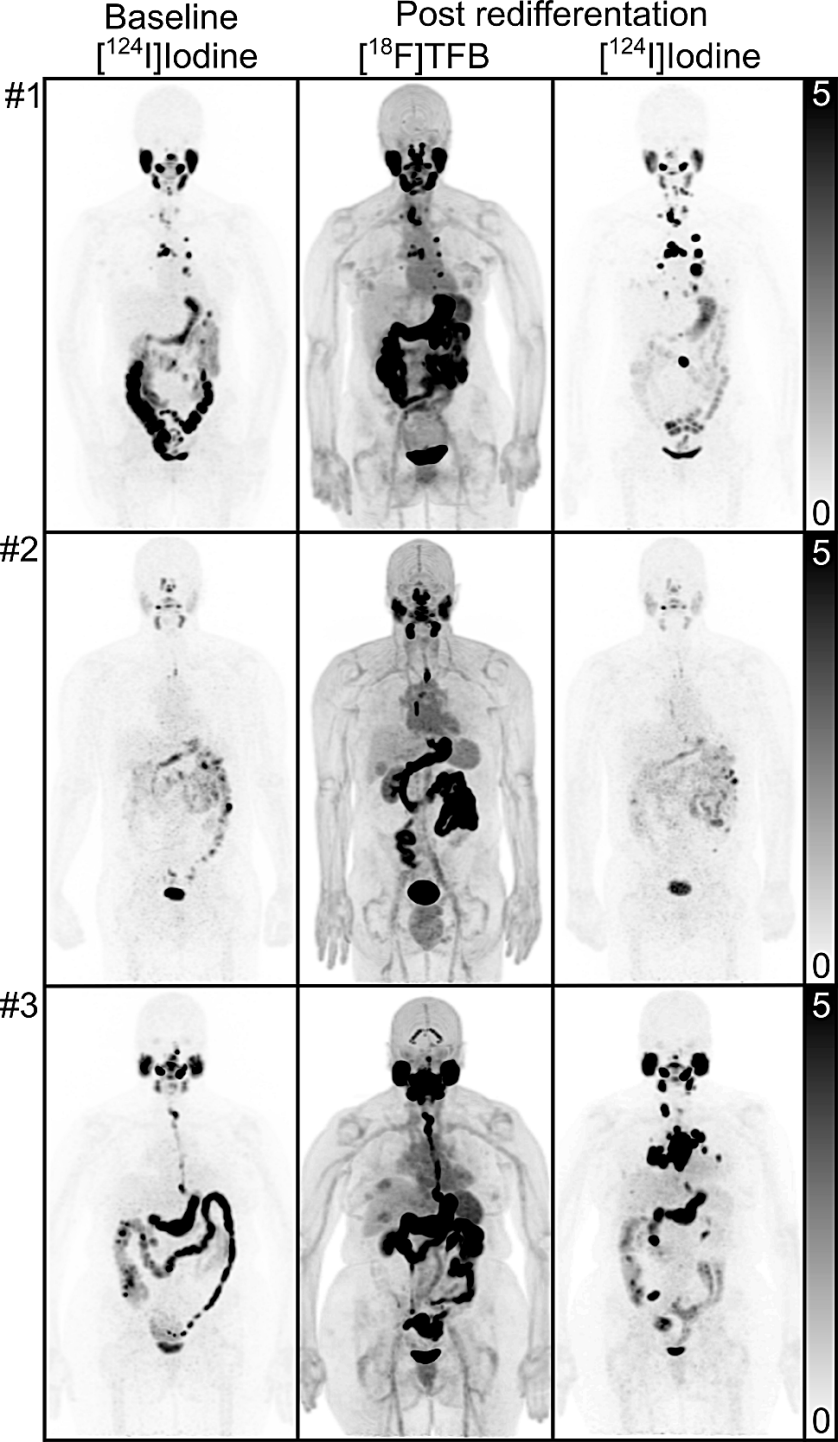
Maximum-intensity projections (MIP) at baseline 48 h [^124^I]Iodine PET/CT (left column), post-redifferentiation [^18^F]TFB PET/CT (middle column), and near-simultaneous post-redifferentiation 48 h [^124^I]Iodine PET/CT for patients #1–3. **Upper row**,** patient #1**: Mostly concordant uptake at neck, mediastinal, lung, and lumbar paraspinal musculature metastases of baseline [^124^I]Iodine and post-redifferentiation [^18^F]TFB PET/CT. In contrast, post-redifferentiation [^124^I]Iodine PET/CT demonstrated higher intensity and more iodine-positive lesions, particularly with regard to lung metastases. **Middle row**,** patient #2**: Mild uptake in upper mediastinal node and faint uptake in lung nodule visible in baseline [^124^I]Iodine (not readily seen in MIP) did not increase with redifferentiation therapy. Metastases were not [^18^F]TFB-avid. **Lower row**,** patient #3**: Morphologically readily visible neck, mediastinal, lung, and sacral bone metastases were neither [^124^I]Iodine-avid at baseline, nor [^18^F]TFB-avid after redifferentiation therapy. In contrast, post-redifferentiation [^124^I]Iodine PET/CT demonstrated intense uptake in metastases with SUV_max_ up to 285.0 in mediastinal nodes. All images are displayed as SUV 0–5

**Fig. 3:**
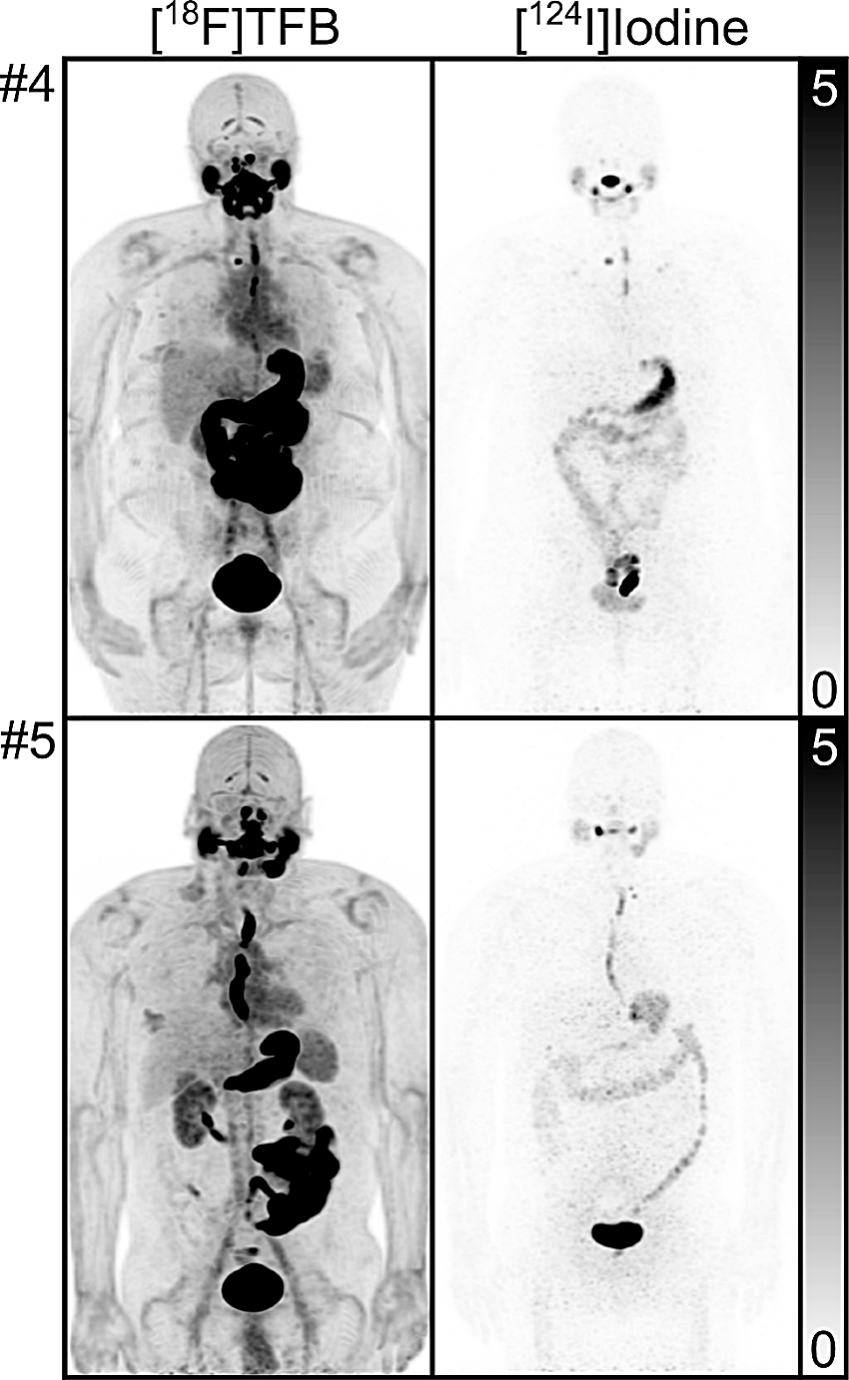
Maximum-intensity projections (MIP) of near-simultaneous [^18^F]TFB PET/CT (left column) and 48 h [^124^I]Iodine PET/CT (right column) for patients #4–5. **Upper row**,** patient #4**: Patient featured concordant lesions as a right lower neck nodule, as well as [^18^F]TFB-positive, [^124^I]Iodine-negative lower lung metastases (not readily visible on MIP; see Figs. 4) and [^18^F]TFB-negative, [^124^I]Iodine-positive upper lung metastases. **Lower row**,** patient #5**: This patient featured mildly [^124^I]Iodine-avid neck nodes without [^18^F]TFB avidity. All images are displayed as SUV 0–5

The normal distribution observed in [^18^F]TFB scanning generally matched the observations of prior studies. Additionally, normal uptake variants and uptake of benign disease were observed that did not feature a correlate in [^124^I]Iodine PET (Figs. 2 and 3): uptake in the choroid plexus was seen in 5/5 patients with median SUV_max_ of 3.9 (range, 1.6-6.0) and in the gall bladder fundus in 4/4 patients without cholecystectomy with median SUV_max_ of 3.2 (range, 2.0-3.5). Moreover, we observed mild uptake in a tentorial meningioma (SUV_max_ 1.6) in patient 1, in hepatic hemangiomas in patient 3 (SUV_max_ 3.4), and in gynecomastia (SUV_max_ 3.1) in patient 5. Concordantly with [^124^I]Iodine scanning, we observed intense uterine fundus uptake in both female patients < 50 years of age (patients 1 and 4, [^18^F]TFB SUV_max_ 10.7 and 24.0, respectively).

Overall, 81 lesions consistent with thyroid cancer metastases were detected in the 5 patients either by [^18^F]TFB or near-simultaneous [^124^I]Iodine PET/CT. Between 2 and 44 lesions were detected in the individual patients. Maximum-intensity projections (MIP) of baseline [^124^I]Iodine and follow-up [^18^F]TFB and near-simultaneous [^124^I]Iodine PET/CTs in patients #1–3 are shown in Fig. 2 and for patients #4–5 in Fig. 3.

[^18^F]TFB-PET detected a total of 19 lesions in 2 patients (patients 1 and 4), resulting in a detection rate of 23%. Lesions’ median SUV_max_ was 4.4 (range, 2.6–13.2). Six neck lesions were detected in two patients with median SUV_max_ of 5.1 (range, 2.8–13.2), nine lung lesions in two patients with median SUV_max_ of 3.6 (range, 2.6–9.2), three mediastinal and hilar nodes in one patient with median SUV_max_ of 5.2 (range, 4.4–5.4) and one soft tissue lesion with SUV_max_ of 5.1.

Near-simultaneous [^124^I]Iodine-PET detected a total of 78 lesions in 5 patients, resulting in a detection rate of 96%. Lesions’ median SUV_max_ was 3.3 (range, 0.4–285.0). Of these, 20 neck lesions were detected in four patients with median SUV_max_ of 2.9 (range, 0.8–21.0), 34 lung lesions in four patients with median SUV_max_ of 1.0 (range, 0.4–36.2), 20 mediastinal and hilar nodes in three patients with median SUV_max_ of 16.1 (range, 1.6–285.0), and 4 bone and soft tissue lesions in two patients with median SUV_max_ of 6.6 (range 2.2–29.5).

**Fig. 4:**
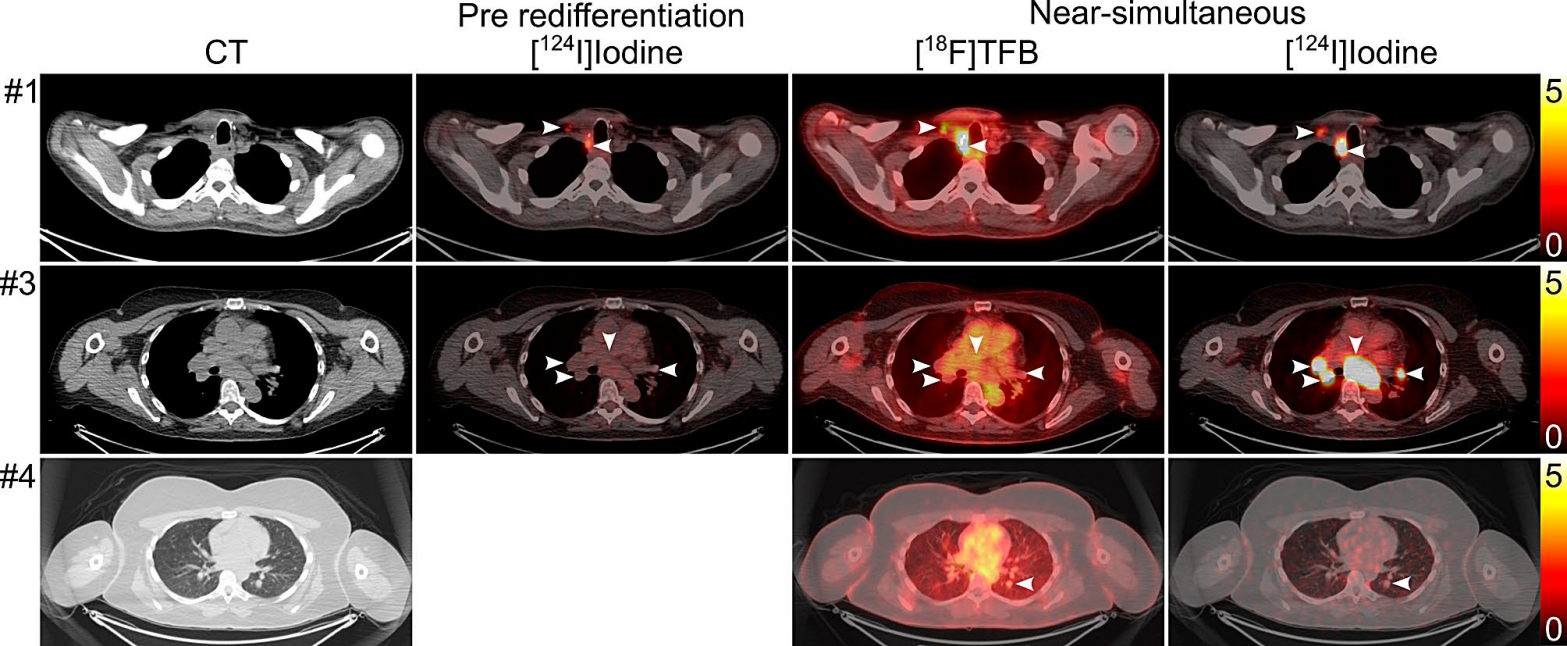
First and second row: transversal CT (first column), corresponding pre-redifferentiation [^124^I]Iodine PET/CT fusion (if available, second column) and after near-simultaneous tracer application, [^18^F]TFB PET/CT fusion (third column), and [^124^I]Iodine PET/CT fusion (fourth column). **First row**, patient 1, example of concordant lesions: Right paratracheal local relapse and right neck nodal metastasis (arrowheads) demonstrate increased [^124^I]Iodine uptake after redifferentiation therapy. Both lesions were well visible in [^18^F]TFB. **Second row**, patient 3, example of better lesion detection with [^124^I]Iodine: At baseline non-[^124^I]Iodine-avid mediastinal, hilar, and lung metastases demonstrated intense uptake after redifferentiation therapy with SUV_max_ up to 285.0 (arrowheads). Lesions did not demonstrate tracer uptake above blood pool in near-simultaneous post-redifferentiation [^18^F]TFB PET/CT. **Third row**, patient 4, example of better lesion detection with [^18^F]TFB: A left lower lobe lung metastasis well visible in CT did not demonstrate [^124^I]Iodine uptake above background noise, but featured mild focal [^18^F]TFB uptake. All images are displayed as SUV 0–5

**Fig. 5:**
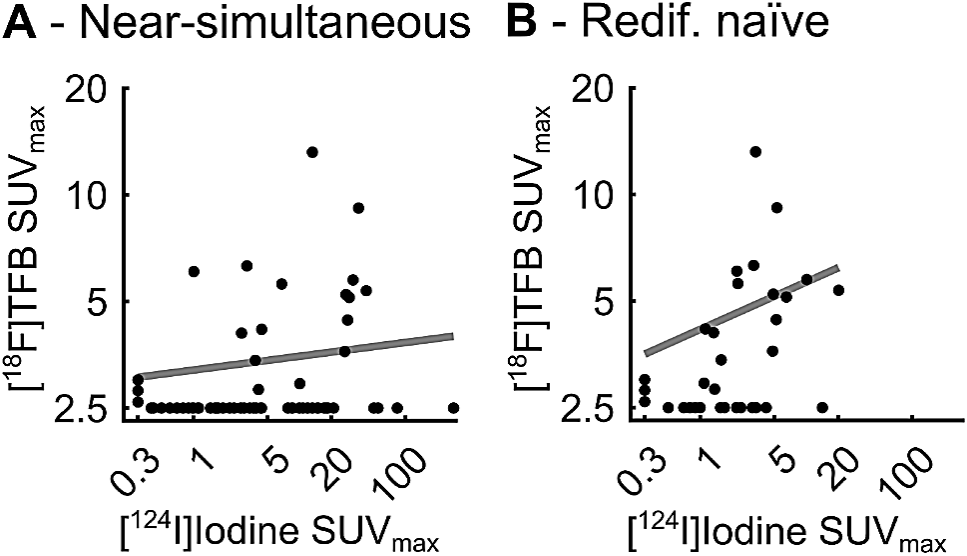
Lesion-based correlation of [^18^F]TFB and [^124^I]Iodine SUVmax either considering near-simultaneous [^124^I]Iodine PET/CT in all 5 patients (**A**) or [^124^I]Iodine PET/CT before (patients 1–3) or without (patients 4–5) redifferentiation therapy (redif. naïve) (**B**). Log-log plots. Trendlines are displayed in gray. Non-discernible lesions’ SUVmax were set to the SUVmax of the lowest detected metastasis minus 0.1 (2.5 for [^18^F]TFB and 0.3 for [^124^I]Iodine) to allow visualization and calculation of correlation coefficients

Sixteen lesions in two patients were positive on [^18^F]TFB and [^124^I]Iodine-PET/CT (Fig. 4, top row). [^18^F]TFB identified three lesions not seen on [^124^I]Iodine imaging in one patient with papillary thyroid cancer (SUV_max_ of 2.6, 2.8, and 3.0; Fig. 4, bottom row). [^124^I]Iodine identified 62 lesions in all 5 patients not seen on [^18^F]TFB with a median SUV_max_ of 2.2 (range, 0.4–285.0) (Fig. 4, middle row). Only a weak correlation between lesion SUV_max_ was observed between [^18^F]TFB and near-simultaneous [^124^I]Iodine (*R* = 0.27, 95% CI: 0.06–0.46; Fig. 5A).

### Comparison of [^18^F]TFB and [^124^I]Iodine-PET/CT before/without redifferentiation therapy

Comparing [^124^I]Iodine PET/CTs before (patients 1–3) or without (patients 4–5) redifferentiation therapy to [^18^F]TFB PET/CT, a total of 33 lesions were seen in 4 patients. This resulted in a [^18^F]TFB detection rate of 58% (19/33). In [^124^I]Iodine PET/CT before/without redifferentiation, 30 lesions in 4 patients with a median SUV_max_ of 2.2 (range, 0.5–20.3) were seen, resulting in a detection rate of 91%. Fourteen [^124^I]Iodine-positive / [^18^F]TFB-negative metastases featured a median [^124^I]Iodine SUVmax of 1.6 (range, 0.5–14.2). A moderate correlation was observed (*R* = 0.64, 95% CI: 0.47–0.76; Fig. 5B) between [^18^F]TFB and [^124^I]Iodine lesional SUV_max_ before/without redifferentiation, superior to the correlation of [^18^F]TFB and near-simultaneous [^124^I]Iodine PET/CT.

### Comparison of [^18^F]TFB and [^124^I]Iodine-PET/CT after redifferentiation therapy

A total of 48 lesions in two patients were newly seen on [^124^I]Iodine PET/CT after re-differentiation therapy with a median SUV_max_ of 3.3 (range, 0.4–285.0). None of these lesions were seen in near-simultaneous [^18^F]TFB scanning.

## Discussion

We report the results of a clinical pilot study exploring the use of [^18^F]TFB PET/CT in patients with poorly differentiated thyroid cancer who underwent redifferentiation therapy or papillary thyroid cancer, in comparison to [^124^I]Iodine PET/CT. After evaluation in small animals [12] and primates [13], biodistribution, safety, and dosimetry of [^18^F]TFB were evaluated in healthy human subjects [14] and patients with thyroid cancer [10]. Clinical comparison to established agents for thyroid cancer imaging is so far limited to retrospective studies from two German centers: Dittmann et al. compared [^18^F]TFB imaging to diagnostic activity [^131^I]Iodine whole-body scanning and SPECT/CT in 21 patients and found higher sensitivity and accuracy of [^18^F]TFB [15]. Ventura et al. from the same center compared [^18^F]TFB imaging to therapeutic activity [^131^I]Iodine whole-body scanning and SPECT/CT in 26 patients and found overall few more [^18^F]TFB-avid than [^131^I]Iodine avid lesions, but also observed true positive [^131^I]Iodine and absent [^18^F]TFB uptake in three patients [16]. Samnick et al. retrospectively evaluated 9 patients with available [^18^F]TFB and 24 h [^124^I]Iodine PET/CT and found that [^18^F]TFB was not inferior to [^124^I]Iodine and even detected [^124^I]Iodine-negative metastases in 2 of 9 patients [17].

In our study sample, in patients with poorly differentiated thyroid cancer, [^18^F]TFB failed to delineate any of the 48 metastases that demonstrated new [^124^I]Iodine uptake after redifferentiation therapy, including most avid metastases with SUV_max_ of up to 285.0. Comparing [^18^F]TFB and [^124^I]Iodine before / without redifferentiation, [^124^I]Iodine again indicated more metastases than [^18^F]TFB, but the discrepancy was less striking and [^18^F]TFB even highlighted a few metastases not seen with [^124^I]Iodine.

Biological and technical aspects must be considered to explain the discrepancies between iodine and [^18^F]TFB scanning and between our findings and those of prior clinical studies. With regard to technical aspects, our study features differences that explain a certain bias toward a more favorable imaging readout of [^124^I]Iodine. The study by Dittmann et al. [15]. used predominantly pretherapeutic administered activities with a median of 315 MBq [^131^I]Iodine for whole-body planar and part-body SPECT/CT. A recent study reported wide agreement of [^124^I]Iodine PET scanning and *therapeutic* activity (median 3.0 GBq) [^131^I]Iodine scanning and SPECT/CT [18]. However, the inferiority of *pretherapeutic* administered activities as used by Dittmann et al. in comparison to scanning with therapeutic [^131^I]Iodine activities or [^124^I]Iodine PET/CT is well established [4, 19]. Ventura et al. compared to therapeutic [^131^I]Iodine activities and as in our study found both exclusively [^131^I]Iodine positive as well as exclusively [^18^F]TFB positive lesions. However, the non-quantitative nature of [^131^I]Iodine scanning prohibited a comprehensive comparison of [^18^F]TFB and [^131^I]Iodine uptake intensity [16]. As in our study, Samnick et al. [17] used [^124^I]Iodine scanning, but we imaged at a later timepoint (48 h as opposed to 24 h), applied more than seven-fold more activity, and invested nearly two-fold more scanning time per bed position. The resulting differences in statistical noise are well appreciable by visual comparison of MIPs from our study and Samnick et al. Moreover, the TFB scans in our study were started at median 60 min p.i., whereas scans were initiated 40 min p.i. in the Dittmann and Samnick studies. However, the study by Ventura et al. demonstrated that uptake at 40 min and 90 min p.i. was highly correlated making the differences in scanning time points an unlikely explanation [16]. Theoretically, false positive foci of Iodine uptake could explain some of the discrepancies observed. In absence of a pathology standard of truth, this cannot be ruled out with certainty for each individual lesion. However, we can certainly exclude a relevant contribution to the overall 81 lesions seen in the analysis. On one hand, false positive Iodine uptake is seldom and can usually be readily identified by companion or correlative imaging [20]. Moreover, most metastases seen in [^124^I]Iodine PET/CT were only seen after redifferentiation therapy, but not in baseline [^124^I]Iodine scanning. Furthermore, lesions evaluated as metastases either had a typical structural correlate or appeared in compartments otherwise unequivocally affected by metastatic disease.

Some of these non-negligible technical differences may account for the moderate discrepancies between [^18^F]TFB and [^124^I]Iodine before / without redifferentiation in comparison to the above studies. However, they cannot explain the very high underestimation of metastatic iodine uptake seen in the patients with poorly differentiated thyroid cancer after redifferentiation therapy with trametinib. This points to an explanation hypothesis building on the pharmacokinetic differences of [^124^I]Iodine and [^18^F]TFB and differences in the underlying biology of differentiated thyroid cancer and poorly differentiated cancer after redifferentiation therapy. [^18^F]TFB is a substrate of NIS, but not of other downstream proteins/enzymes involved in organification as TG or TPO. In the patients with poorly differentiated thyroid cancer, trametinib may have predominantly impacted re-expression of downstream enzymes/proteins such as TPO and TG, which are more relevant for iodine retention and had a lesser or no effect on NIS expression. Vice versa, higher NIS and lower downstream enzyme expression may account for the few observed metastases positive in [^18^F]TFB and negative in [^124^I]Iodine.

Our study has limitations, particularly the small cohort of only five patients with different thyroid cancer types and different therapy regimens, allowing us to establish hypotheses, but not to reach definite conclusions. Furthermore, although iodine dosimetry scanning protocols were applied in every patient, the abbreviated nature of the protocols in some patients did not allow to include consistent dosimetry values for individual metastases in the analysis.

## Conclusions

In conclusion, [^18^F]TFB failed to predict radioactive iodine uptake in patients with poorly differentiated thyroid cancer who underwent redifferentiation therapy with trametinib. It is unclear whether such discrepancies may also occur in other redifferentiation therapies or if they may even be relevant in redifferentiation-naïve differentiated thyroid cancer.

## Data Availability

The datasets used and/or analyzed during the current study are available from the corresponding author on reasonable request.

## Abbreviations

DTC: Differentiated thyroid cancer
Et2OBF3: Boron trifluoride diethyl etherate
[^18^F]: Fluorine-18
MIP: Maximum-intensity projection
NaCl: Sodium chloride
NIS: Sodium iodine symporter
RIT: Radioiodine therapy
SUV: Standardized uptake value
Tc-99 m: Technetium-99 m
TG: Thyroglobulin
TFB: Tetrafluoroborate
TPO: Thyroperoxidase
TSH: Thyroid-stimulating hormone
